# Risk prediction for symptomatic ischemic cerebrovascular disease based on ultrasound indicators of carotid plaque neovascularization

**DOI:** 10.3389/fcvm.2025.1648352

**Published:** 2025-10-06

**Authors:** Jianmei Chen, Jia Wang, Qiushuang Wang, Wenqi Sun, Xiaoyan Huo, Xinna Li

**Affiliations:** ^1^ Department of Health Medicine, The Fourth Medical Center of Chinese PLA General Hospital, Beijing, China; ^2^ Department of Ultrasound Diagnostics, The Second Affiliated Hospital of Air Force Medical University, Xi'An, China; ^3^ Department of Ultrasound Diagnostics, The Air Force Medical Center, Air Force Medical University, Beijing, China; ^4^ Diagnostic Ultrasound Department, The Sixth Medical Center of PLA General Hospital, Beijing, China; ^5^ Medical Technology Support Department, The PLA General Hospital Jingzhong Medical Area, Beijing, China

**Keywords:** automated machine learning (AutoML), symptomatic ischemic cerebrovascular disease (symptomatic ICVD), carotid plaque, prediction model, risk factors, contrast-enhanced ultrasound (CEUS)

## Abstract

**Objective:**

To construct a model for predicting the risk of symptomatic ischemic cerebrovascular disease (ICVD) based on carotid plaque characteristics utilizing Automated Machine Learning (AutoML) technology, systematically identify key predictive factors, and provide evidence for clinical risk stratification and individualized intervention.

**Methods:**

A single-center retrospective study design was employed, enrolling 626 patients with carotid plaques who were treated between January 2020 and December 2022. Structured electronic medical records (EMRs) were used to extract comprehensive clinical data, including: Demographic characteristics (gender, age); Cardiovascular risk factors (e.g., hypertension, diabetes mellitus); Lifestyle habits (smoking, alcohol consumption); Laboratory parameters (blood lipid profiles, C-reactive protein); Ultrasound-evaluated carotid plaque characteristics (stenosis severity, ulcer formation, plaque number, intraplaque neovascularization). The dataset was divided into a training set (501 patients, ∼80%) and a test set (125 patients, ∼20%). Utilizing the AutoML framework, we implemented the Improved Newton-Raphson Based Optimizer (INRBO) to optimize model hyperparameters. Feature importance was validated through dual-dimensional analysis employing LASSO regression and SHAP (SHapley Additive exPlanations) interpretability models. Furthermore, an interactive nursing decision support system was developed using MATLAB.

**Results:**

Among the 626 patients, 375 (59.90%) developed symptomatic ICVD. The prediction model constructed in this study demonstrated significantly enhanced performance: On the training set: ROC-AUC rose to 0.9537 and PR-AUC improved to 0.9522. On the independent test set: ROC-AUC remained high at 0.9343 and PR-AUC was 0.9104. These results consistently surpassed all other comparative models. The model definitively identified six core variables predicting symptomatic ICVD onset: Stenosis Severity; Ulcerative Plaque; Plaque Number; Intraplaque Neovascularization; Age; Diabetes Status. LASSO regression analysis independently selected seven variables, achieving an 85.71% overlap rate (6 out of 7 features) with the features selected by the AutoML model. SHAP analysis confirmed the top three feature importance rankings: (1) Stenosis Severity, (2) Ulcerative Plaque, (3) Plaque Number.

**Conclusion:**

By integrating multidimensional clinical data with interpretable machine learning techniques, this study confirms the pivotal role of carotid plaque morphological features and metabolic factors in symptomatic ICVD risk prediction. Crucially, it achieves the real-time translation of risk assessment into actionable intervention decisions, thereby providing innovative tools and methodological advances for the precision diagnosis and treatment of cerebrovascular diseases.

## 1 Introduction

Ischemic cerebrovascular disease (ICVD) is one of the leading global causes of disability and mortality. Among its forms, symptomatic ICVD poses a core clinical challenge in neurology due to its high incidence, elevated recurrence rate, and poor prognosis (1). The formation of carotid atherosclerotic plaques is widely recognized as a key pathological basis for triggering symptomatic ICVD. Its pathogenesis is closely linked to the biological characteristics of the plaques, particularly intraplaque neovascularization (IPN), which serves as a core marker for assessing plaque vulnerability (2, 3). Molecular biology studies indicate that IPN promotes erythrocyte extravasation and inflammatory cell infiltration by secreting vascular endothelial growth factor (VEGF) and matrix metalloproteinases (MMPs). This process subsequently weakens the structural stability of the fibrous cap, ultimately triggering plaque rupture and thromboembolic events (4, 5). More significantly, prospective cohort studies have confirmed that IPN is not only an independent predictor of recurrent events within one month in acute stroke patients but also significantly correlates with the risk of coronary artery disease, highlighting its cross-system value in predicting systemic vascular events (6).

In recent years, multimodal imaging techniques have been extensively applied for the non-invasive evaluation of carotid plaques. Computed tomography angiography (CTA), magnetic resonance angiography (MRA), digital subtraction angiography (DSA), and positron emission tomography (PET) can precisely analyze plaque morphological and functional characteristics. The current landscape of carotid plaque assessment primarily features digital subtraction angiography (DSA) as the gold standard for evaluating stenosis severity and plaque ulceration (7). However, DSA demonstrates critical limitations in characterizing plaque composition due to its fundamental methodological constraints: As a luminographic technique, it visualizes blood flow dynamics but lacks the resolution to differentiate intraplaque components such as lipid-rich necrotic cores, fibrous cap integrity, or intraplaque hemorrhage. This limitation is compounded by its invasive nature—requiring arterial puncture—which carries procedural risks including hematoma formation, vascular dissection, and cerebral embolism. Alternative modalities like CT angiography suffer from radiation exposure limitations, while MRI faces accessibility barriers due to prolonged scan times and high costs (8, 9). Collectively, these constraints highlight an urgent need for non-invasive, high-resolution techniques capable of comprehensive plaque characterization. In contrast, conventional ultrasound is widely utilized for preliminary assessment of morphological parameters like plaque thickness and echogenicity due to its advantages of being non-invasive, low-cost, and enabling real-time dynamic imaging. Nevertheless, its limited spatial resolution hinders the identification of micrometer-scale neovascular structures (10).

The innovative application of contrast-enhanced ultrasound (CEUS) offers a new pathway to overcome these bottlenecks (11). This technology non-invasively visualizes neovessels with diameters <100 μm by intravenous injection of inert gas microbubbles encapsulated by phospholipid shells, leveraging their nonlinear oscillation properties to enhance blood flow signals. Additionally, the pulmonary metabolism mechanism of the microbubbles within the body ensures circulatory stability. However, translating imaging features into clinical risk prediction still faces a critical obstacle: the insufficient efficacy of existing risk models. Traditional prediction tools suffer from two major deficiencies: firstly, their sensitivity and ROC-AUC are generally low, making it difficult to effectively stratify high-risk populations; secondly, the underlying algorithms are prone to issues like sensitivity to initial values and local convergence, often leading optimization processes to suboptimal solutions, thereby compromising model robustness and generalizability (12). Enhanced intelligent algorithms and machine learning methods demonstrate significant advantages in constructing predictive models based on multi-source data (e.g., imaging, clinical and demographic features) (13, 14). These intelligent algorithms can integrate high-dimensional heterogeneous variables and capture complex non-linear relationships, thereby optimizing the early identification accuracy for symptomatic ICVD risk, and providing support for personalized intervention strategies. Currently, the predictive value of intraplaque neovascularization for symptomatic ICVD occurrence and its associated risk factors remain unclear.

Our study employs an integrative artificial intelligence framework to build a multidimensional prediction model for symptomatic ischemic cerebrovascular disease (ICVD). While CEUS comprehensively characterizes plaque morphology—including features such as intraplaque hemorrhage, ulceration, and neovascularization—this work specifically centers on intraplaque neovascularization (IPN) as the core imaging biomarker for three key reasons: (1) IPN is a well-established histopathological hallmark of plaque vulnerability, strongly correlated with future cardiovascular events; (2) CEUS uniquely enables quantitative assessment of microvascular perfusion (<100 μm resolution) non-invasively; and (3) IPN features demonstrate superior dynamic range for algorithmic learning, as evidenced by recent radiomics literature. Crucially, our AI framework synthesizes IPN quantification with 24 complementary variables—spanning conventional ultrasound markers (plaque thickness, echogenicity, stenosis degree), clinical risk factors, and biochemical profiles—to achieve holistic risk stratification beyond any single modality.

## 2 Methods

### 2.1 Study population

This single-center retrospective study enrolled patients diagnosed with carotid artery plaques by vascular ultrasound at the Fourth Medical Center of the PLA General Hospital between January 2020 and December 2022. As a retrospective analysis, patient informed consent was waived. The study protocol was reviewed and approved by the Ethics Committee of the Fourth Medical Center of PLA General Hospital (Ethics Approval Number: LC-202505175).

Inclusion criteria were: (1) Meeting the diagnostic criteria for ICVD; (2) Detection of carotid atherosclerotic plaque by conventional ultrasound and subsequent undergoing of contrast-enhanced ultrasound (CEUS); (3) Full consciousness.

Exclusion criteria were: (1) Major organ dysfunction; (2) Immune system disorders or malignancies; (3) Known allergy to ultrasound contrast agents; (4) Contraindications to ultrasound examination; (5) Poor quality ultrasound images.

### 2.2 Data collection

All patient data were extracted from the hospital's electronic medical record (EMR) system using a structured approach. The collected data encompassed: demographic characteristics (gender, age), cardiovascular risk factors (hypertension, hyperlipidemia, diabetes mellitus), lifestyle habits (smoking, alcohol consumption), laboratory parameters (lipid profiles, fasting blood glucose, C-reactive protein), and carotid plaque characteristics assessed by ultrasound. All patients underwent standardized ultrasound examinations performed by two certified sonographers using a Siemens ACUSON Sequoia system (9L4 transducer). Plaque echogenicity was visually classified during live B-mode imaging according to Gray-Weale criteria as: hyperechoic, isoechoic, hypoechoic, or anechoic. Contrary to research-focused quantitative texture analysis tools, this clinical assessment relied on real-time sonographer interpretation rather than offline computerised plaque analysis software. The echogenicity assessment followed established clinical protocols where plaques were categorized as “low echogenicity” if exhibiting hypoechoic or anechoic characteristics, consistent with current guideline-recommended practice (15). All sonographers underwent pre-study calibration training to standardize interpretation, with inter-observer agreement validated through independent assessments of 50 random cases (*κ* = 0.82). Data quality control was implemented through dual-independent data entry and logic validation. Discrepancies were resolved by arbitration from a third senior expert. Patients missing baseline data or with substandard image quality were excluded during case screening. Ultimately, 626 patients were included. The outcome variable was whether the patient had symptomatic ICVD. Symptomatic ICVD was defined as: the presence of new-onset focal neurological deficit symptoms (e.g., hemiplegia, aphasia, visual field loss) persisting for >24 h, confirmed by CT/MRI or digital subtraction angiography (DSA) as acute cerebral infarction or an ischemic lesion within the territory of the responsible artery, and independently reviewed and confirmed by at least two associate chief physicians of neurology. Patients with transient ischemic attacks (TIA) or asymptomatic imaging findings of ischemic lesions were excluded from the symptomatic ICVD group.

The presence or absence of IPN served as a key predictive feature in this study. To ensure objective and standardized assessment, IPN was evaluated using contrast-enhanced ultrasound (CEUS) examinations, focusing specifically on the detection of microbubbles (signal enhancement) within the carotid plaque, indicative of neovessel formation and permeability. Definition of IPN Presence: IPN was strictly classified as a binary variable (present/absent) based on established CEUS criteria and visual interpretation by experienced readers:

Presence (“Yes”): Defined as the visualized penetration and accumulation of microbubbles within the carotid plaque substance during the dynamic contrast phase. This was confirmed by the observation of discrete, punctate, or linear enhancement signals originating within the plaque core on CEUS cine loops, persisting for several seconds. Enhancement confined solely to the plaque surface, shoulders, or the adventitia, without clear evidence of intraplaque penetration, was not classified as IPN presence.
Absence (“No”): Defined as the lack of any detectable microbubbles within the plaque substance observed throughout the dynamic CEUS examination. Plaque enhancement, if any, was limited to the surface or immediately adjacent adventitial tissues without intraplaque migration.

### 2.3 Model development

Our study proposes an adaptive ensemble modeling framework based on an improved Newton-Raphson-based optimizer (INRBO). The framework employs a 0–1 encoding full-parameter space collaborative optimization strategy to achieve synchronous optimization of feature selection, base classifier configuration, and hyperparameter tuning. During data partitioning, stratified random sampling was applied based on the outcome variable (“symptomatic ICVD” vs. non-symptomatic) to divide the entire dataset into a training set (*N* = 501) and an independent test set (*N* = 125) at a 4:1 ratio, ensuring proportional distribution of outcome classes in both the original cohort and subsets. During data preprocessing, we specifically implemented the Synthetic Minority Oversampling Technique (SMOTE) to address class imbalance in our training cohort (375 symptomatic ICVD cases vs. 251 non-symptomatic, 60:40 ratio). This technique generated synthetic minority class instances through feature space interpolation between neighboring cases prior to model optimization. The SMOTE application was exclusively confined to the training set using its default k-nearest neighbors parameter (*k* = 5) while the independent test set remained untouched to preserve real-world validation integrity.

Addressing the limitations of the traditional Newton-Raphson optimizer (NRO), specifically its susceptibility to initial value sensitivity and local convergence constraints (16), our study enhanced its global search capability through a two-stage improvement: (1) Parameter Initialization: Tent chaotic mapping was employed to generate the initial candidate solution set, improving parameter space coverage. (2) Iterative Update: A dynamic Gaussian mutation mechanism was embedded. When fitness stagnated (no improvement over five consecutive iterations), perturbation was applied to the current best solution via mutation to escape local optima traps. While feature engineering can enhance predictive performance, we prioritized biological interpretability and translational applicability, ensuring model parsimony. Thus, our primary analysis utilized raw clinical features directly extracted from electronic medical records, intentionally preserving clinical feature integrity.

**Figure 1:**
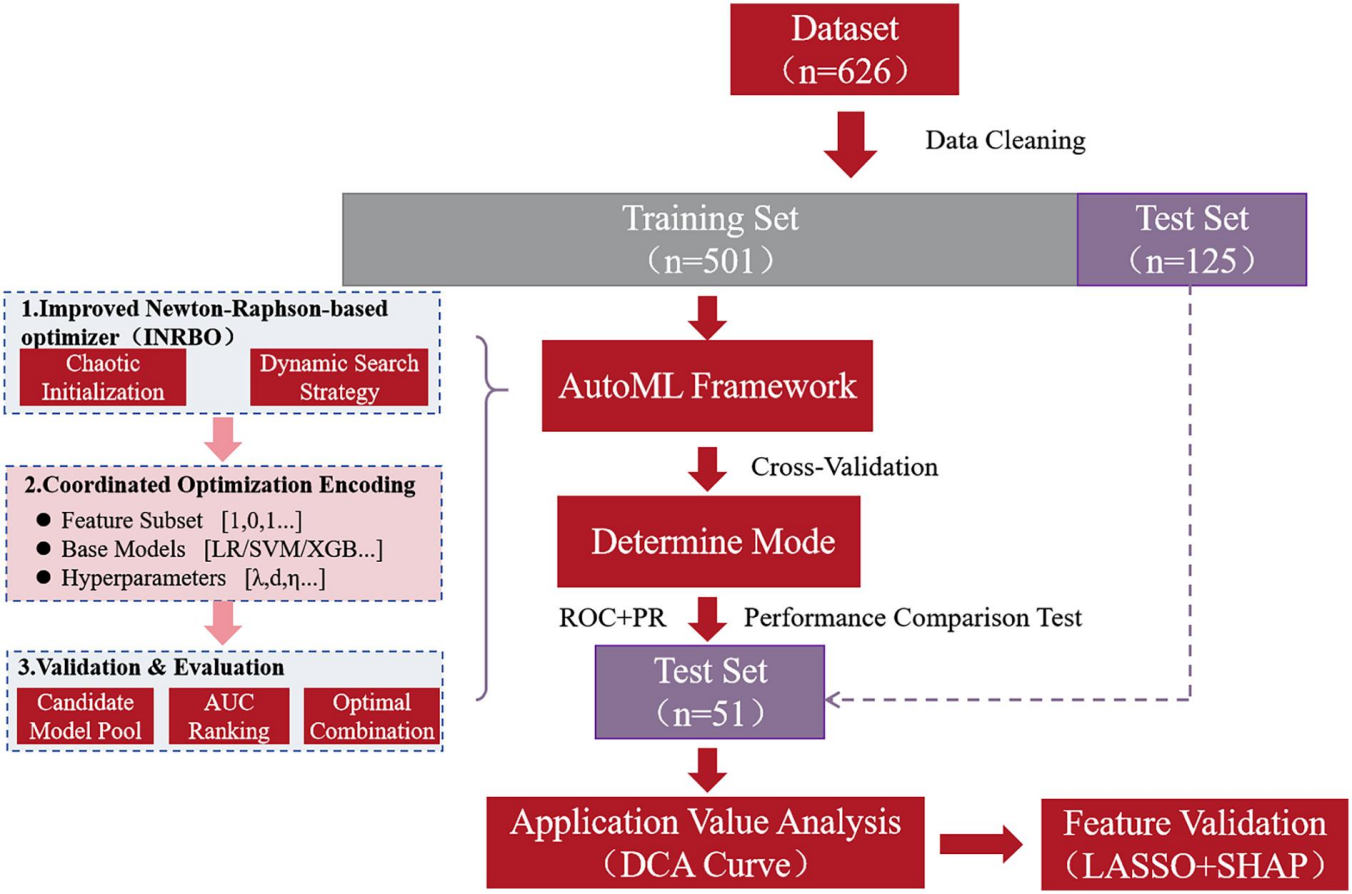
Flow chart of the study.

During model construction, a hybrid encoding scheme (0–1 feature mask vector+base classifier index code + real-valued hyperparameter vector) was designed to establish a unified optimization space. Each generated individual in the INRBO population encoding represented a specific configuration: selected feature subset (1 for inclusion, 0 for exclusion), type of base learner (selected from a heterogeneous model pool comprising Logistic Regression, Support Vector Machine, AdaBoost, XGBoost, and LightGBM), and associated hyperparameter combinations [e.g., regularization coefficient λ ∈ (0.01, 10), maximum tree depth for boosting d ∈ (3, 15), learning rate η ∈ (0.001, 0.3)]. The algorithm's fitness evaluation was driven by the five-fold cross-validated Area Under the Receiver Operating Characteristic Curve (AUC) on the training set. The model's generalization performance was validated using the independent test set. The final output of the framework corresponded to the optimized feature-model-hyperparameter joint configuration. The overall research workflow is depicted in Figure 1.

### 2.4 Evaluation metrics

This study employed multi-dimensional quantitative metrics to evaluate the model's classification performance and clinical utility. Fundamental classification metrics included: Precision (PRE): Measuring the accuracy of positive predictions. Sensitivity (SEN - Recall): Assessing the ability to identify positive-class samples. Specificity (SPE): Reflecting the capacity to exclude negative-class samples. Accuracy (ACC): Representing the overall classification correctness. F1-score: Harmonizing the trade-off between precision (PRE) and sensitivity (SEN). ROC-AUC (Area Under the Receiver Operating Characteristic Curve): Quantifying the model's discriminative power for potential sample categories. PR-AUC (Area Under the Precision-Recall Curve): Evaluating stability specifically for imbalanced datasets. For assessing clinical application value, Decision Curve Analysis (DCA) compared the net benefit of model-guided prediction strategies against baseline intervention scenarios (e.g., intervene-all or intervene-none) across dynamically varying threshold probabilities. This validated the model's effective threshold range and its generalizability for risk assessment in clinical contexts.

### 2.5 Feature validation

The scientific validity and clinical plausibility of prognostic prediction features were rigorously verified through a dual-dimensional approach combining LASSO regression analysis and the SHAP (SHapley Additive exPlanations) interpretability model. First, the LASSO regression algorithm was applied for sparse modeling of high-dimensional clinical features. Its adaptive regularization constraint mechanism effectively eliminated redundant variables, screening out key features significantly associated with symptomatic ICVD patients. This ensured model parsimony and robustness against overfitting. Subsequently, the SHAP interpretability framework was used to elucidate the contribution of selected features at both the global (overall model behavior) and local (individual prediction) levels. SHAP quantified the magnitude and direction (positive or negative influence) of each variable on the patient's predicted risk score. These results were then interpreted in light of clinical prior knowledge to verify the consistency of synergistic or antagonistic interactions among features. This multi-scale approach ultimately revealed complex association patterns between key biomarkers and the clinical endpoint events (symptomatic ICVD), providing transparent visual explanations of the model's predictive logic. This enhanced the interpretability and credibility of the clinical decision support system.

### 2.6 Decision support system development

Using MATLAB's App Designer for interactive application development, we designed and implemented an Intelligent Nursing Decision Support Platform. The core functional module of this system is the integrated prognostic prediction model. The platform provides user interfaces for patient data input, real-time risk probability calculation, and automatic generation of prediction results. Employing a modular design, it supports deployment across different platforms (e.g., web-based or local servers). Graphical controls and dynamic visualization of results enhance human-computer interaction efficiency, providing a reliable technical foundation for precise decision-making in clinical practice.

### 2.7 Statistical analysis

All data were imported into the SPSS 26.0 statistical analysis platform for standardized processing: Continuous variables conforming to a normal distribution were presented as mean ± standard deviation. Categorical variables were presented as percentages. Differences in continuous variables between two groups were analyzed using the independent samples *t*-test. Associations between categorical variables across groups were analyzed using the Chi-square test. A two-sided *p*-value <0.05 was considered statistically significant.

## 3 Results

### 3.1 Baseline characteristics of different datasets

A total of 626 patients were included in our study. Among them, 375 patients (59.90%) experienced symptomatic ICVD. The clinical characteristics of patients in the training set (*N* = 501) and the test set (*N* = 125) are compared below (Table 1). The results demonstrate no statistically significant differences (*p* > 0.05) in any of the clinical characteristics between the two datasets.

**Table 1:** Comparison of clinical characteristics between training Set and test Set.

Characteristic	Training set (*n* = 501)	Test set (*n* = 125)	Statistics	*p*-value
Male, *n* (%)	290 (57.88%)	77 (61.60%)	0.569	0.450
Age (years, Mean ± SD)	69.93 ± 9.61	71.42 ± 10.58	1.519	0.129
BMI (kg/m^2^, Mean ± SD)	26.54 ± 4.24	27.19 ± 5.31	1.453	0.147
Hypertension [Yes, *n* (%)]	364 (72.65%)	93 (74.40%)	0.155	0.694
Hyperlipidemia [Yes, *n* (%)]	297 (59.28%)	71 (56.80%)	0.254	0.614
Diabetes [Yes, *n* (%)]	160 (31.94%)	46 (36.80%)	1.072	0.301
Smoking [Yes, *n* (%)]	385 (76.85%)	101 (80.80%)	0.901	0.343
Alcohol use [Yes, *n* (%)]	311 (62.08%)	71 (56.80%)	1.171	0.279
TC (mmol/L, Mean ± SD)	4.69 ± 0.64	4.80 ± 0.57	1.756	0.08
TG (mmol/L, Mean ± SD)	1.95 ± 0.34	1.98 ± 0.3	0.903	0.367
HDL-C (mmol/L, Mean ± SD)	1.26 ± 0.31	1.25 ± 0.29	0.327	0.744
LDL-C (mmol/L, Mean ± SD)	2.65 ± 0.38	2.72 ± 0.40	1.823	0.069
Fasting blood glucose (mmol/L, Mean ± SD)	5.52 ± 0.55	5.47 ± 0.48	0.932	0.352
C-reactive protein (mg/L, Mean ± SD)	8.37 ± 1.08	8.43 ± 1.03	0.561	0.575
Bilateral mean carotid intima-media thickness (mm, Mean ± SD)	1.12 ± 0.35	1.18 ± 0.40	1.665	0.097
Ulcerative plaque [Yes, *n* (%)]	261 (52.10%)	58 (46.40%)	1.299	0.254
Low echogenicity [Yes, *n* (%)]	275 (54.89%)	66 (52.80%)	0.176	0.675
Intraplaque neovascularization [Yes, *n* (%)]	305 (60.88%)	80 (64.00%)	0.412	0.521
Plaque thickness [*n* (%)]				
<4 mm	291 (58.08%)	67 (53.60%)	0.821	0.365
≥4 mm	210 (41.92%)	58 (46.40%)		
Plaque number [*n* (%)]				
Single	315 (62.87%)	73 (58.40%)	0.85	0.357
Multiple	186 (37.13%)	52 (41.60%)		
Stenosis Degree [*n* (%)]				
<70%	309 (61.68%)	73 (58.40%)	0.452	0.502
≥70%	192 (38.32%)	52 (41.60%)		

### 3.2 Algorithm performance comparison

**Figure 2:**
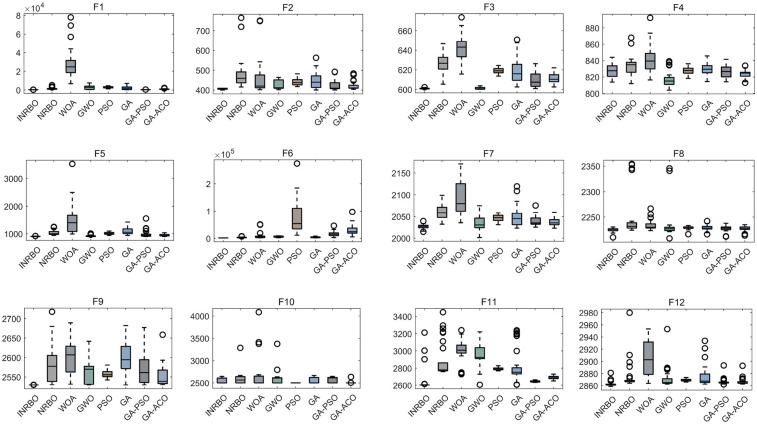
Performance comparison of swarm intelligence optimization algorithms.

**Figure 3:**
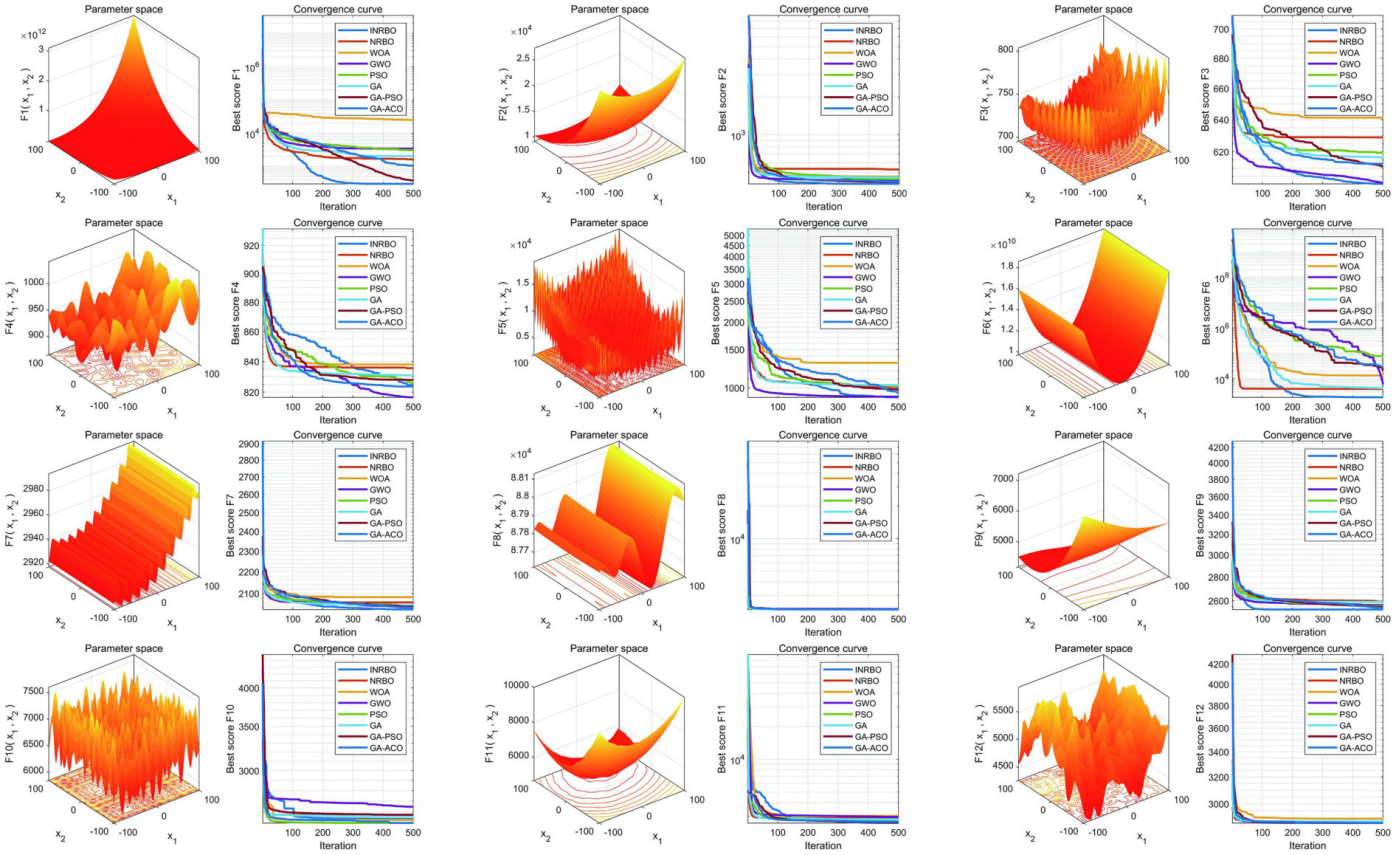
Convergence performance comparison of swarm intelligence optimization algorithms.

To verify the optimization capability of the improved INRBO algorithm, our study conducted comparative tests against the original NRBO, Whale Optimization Algorithm (WOA), Grey Wolf Optimizer (GWO), Particle Swarm Optimization (PSO), Genetic Algorithm (GA), GA-PSO hybrid algorithm, and GA-Ant Colony Optimization (ACO) hybrid algorithm. The experiments utilized all 12 benchmark functions from the CEC2022 test suite. All testing functions were configured with a variable dimension of 10, a population size of 30, and a maximum iteration count of 500. Each algorithm was independently run 30 times to ensure statistical reliability. Based on the 30 independent run outcomes, box plots were generated to assess the optimization stability of each algorithm. The results demonstrated that INRBO consistently outperformed its counterparts in the vast majority of the test functions, showcasing significantly superior stability compared to the original NRBO and the other benchmarked algorithms (Figure 2). Furthermore, convergence curve analysis indicated that INRBO achieved faster convergence speeds and exhibited the lowest risk of becoming trapped in local optima during the iterative process (Figure 3). These experimental findings conclusively substantiate the significant advantages of the INRBO algorithm in terms of global optimization performance and convergence efficiency.

### 3.3 Model training results and key factor identification

**Figure 4:**
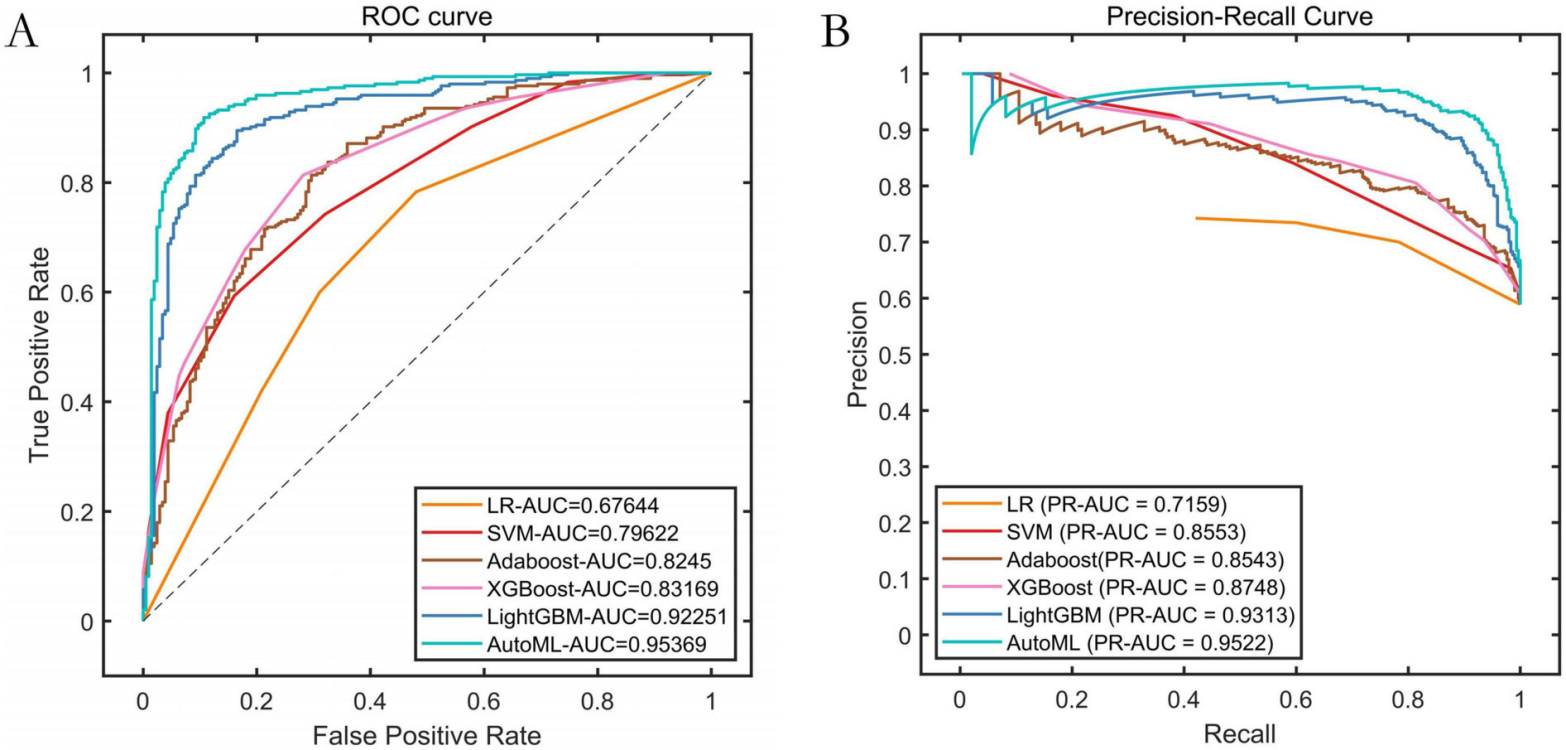
Performance comparison of training set cross-validation. **(A)** ROC curves of the training set; **(B)** Precision-Recall (PR) curves of the training set.

Based on the feature screening results from the improved automatic machine learning framework (INRBO-AutoML), five core variables predictive of symptomatic ICVD were ultimately identified: Stenosis Severity, Ulcerative Plaque, Plaque Number, Intraplaque Neovascularization, and Age. Employing a multi-objective optimization framework (balancing predictive performance with clinical interpretability), the model automatically selected LightGBM as the optimal base learner. Comparative experimental results validated through cross-validation against six groups of traditional machine learning models (Logistic Regression, Random Forest) and traditional ensemble models (AdaBoost, XGBoost, LightGBM) demonstrated that INRBO-AutoML achieved significantly superior performance: It attained an F1-Score of 0.9045. Its ROC-AUC and PR-AUC significantly increased to 0.9537 and 0.9522, respectively. Detailed results are presented in Table 2 and Figure 4.

**Table 2:** Performance of models on the training set (cross-validation).

Models	PRE	SEN	SPE	ACC	F1	ROC-AUC	PR-AUC
LR	0.7000	0.7831	0.5194	0.6747	0.7392	0.6764	0.7159
SVM	0.6909	0.9017	0.4223	0.7046	0.7824	0.7962	0.8553
Adaboost	0.7514	0.8915	0.5777	0.7625	0.8155	0.8245	0.8543
XGBoost	0.8054	0.8136	0.7184	0.7745	0.8094	0.8317	0.8748
LightGBM	0.7871	0.9525	0.6311	0.8204	0.8620	0.9225	0.9313
AutoML	0.8529	0.9627	0.7621	0.8802	0.9045	0.9537	0.9522

### 3.4 Predictive performance on the test set

**Figure 5:**
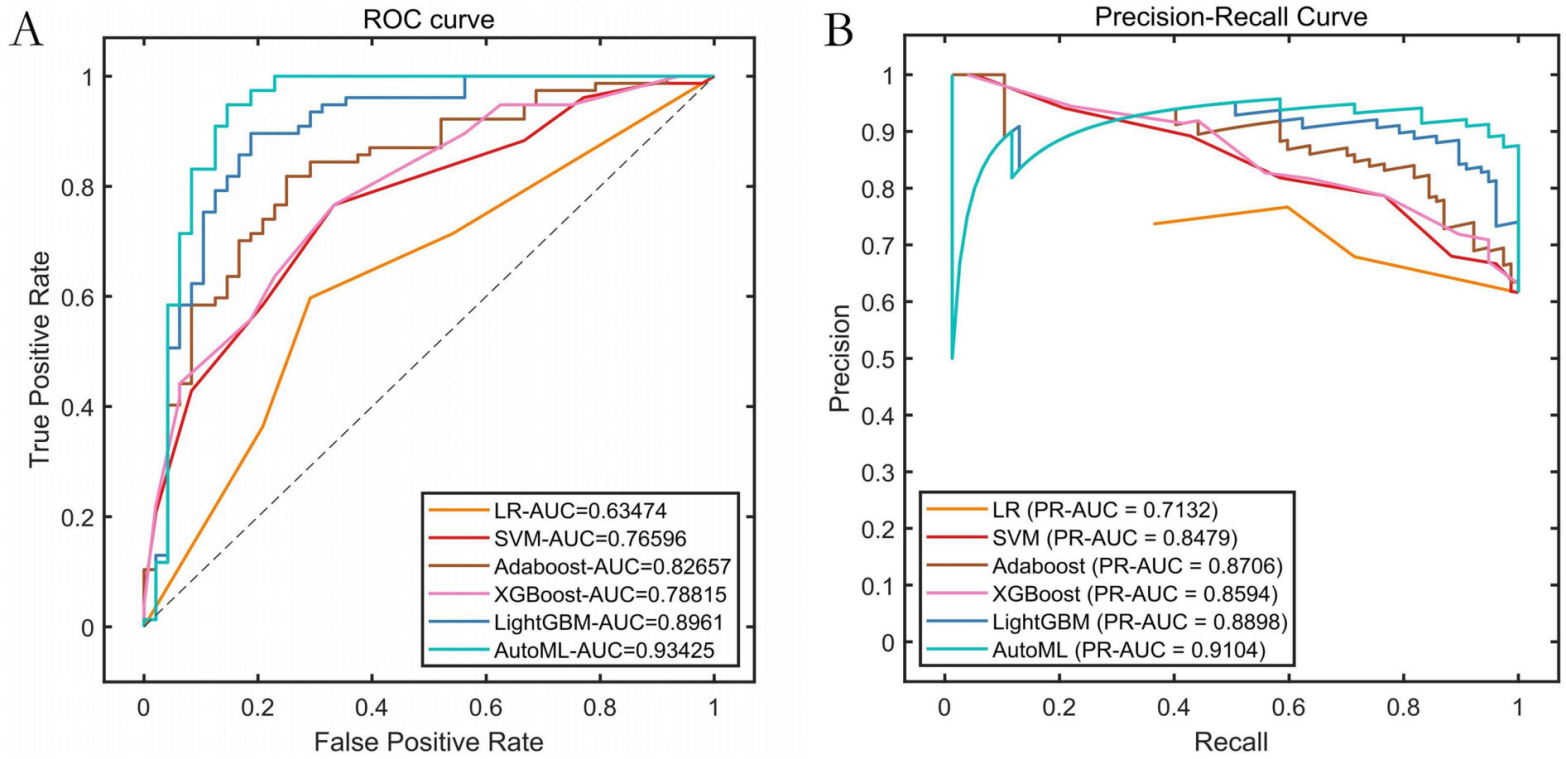
Performance curves. **(A)** ROC curve of the training set; **(B)** Precision-Recall (PR) curve of the training set.

Results on the independent test set confirmed the outstanding predictive capability of the AutoML model: It achieved an F1-Score of 0.9333, ROC-AUC of 0.9343, and PR-AUC of 0.9104. This strong performance validated its excellent generalization ability. Detailed metrics are listed in Table 3 and visualized in Figure 5.

**Table 3:** Model performance on the test set.

Models	PRE	SEN	SPE	ACC	F1	ROC-AUC	PR-AUC
LR	0.6790	0.7143	0.4583	0.6160	0.6962	0.6347	0.7132
SVM	0.6800	0.8831	0.3333	0.6720	0.7684	0.7660	0.8479
Adaboost	0.7701	0.8701	0.5833	0.7600	0.8171	0.8266	0.8706
XGBoost	0.7867	0.7662	0.6667	0.7280	0.7763	0.7882	0.8594
LightGBM	0.8111	0.9481	0.6458	0.8320	0.8743	0.8961	0.8898
AutoML	0.8750	1.0000	0.7708	0.9120	0.9333	0.9343	0.9104

### 3.5 Clinical application value analysis

**Figure 6:**
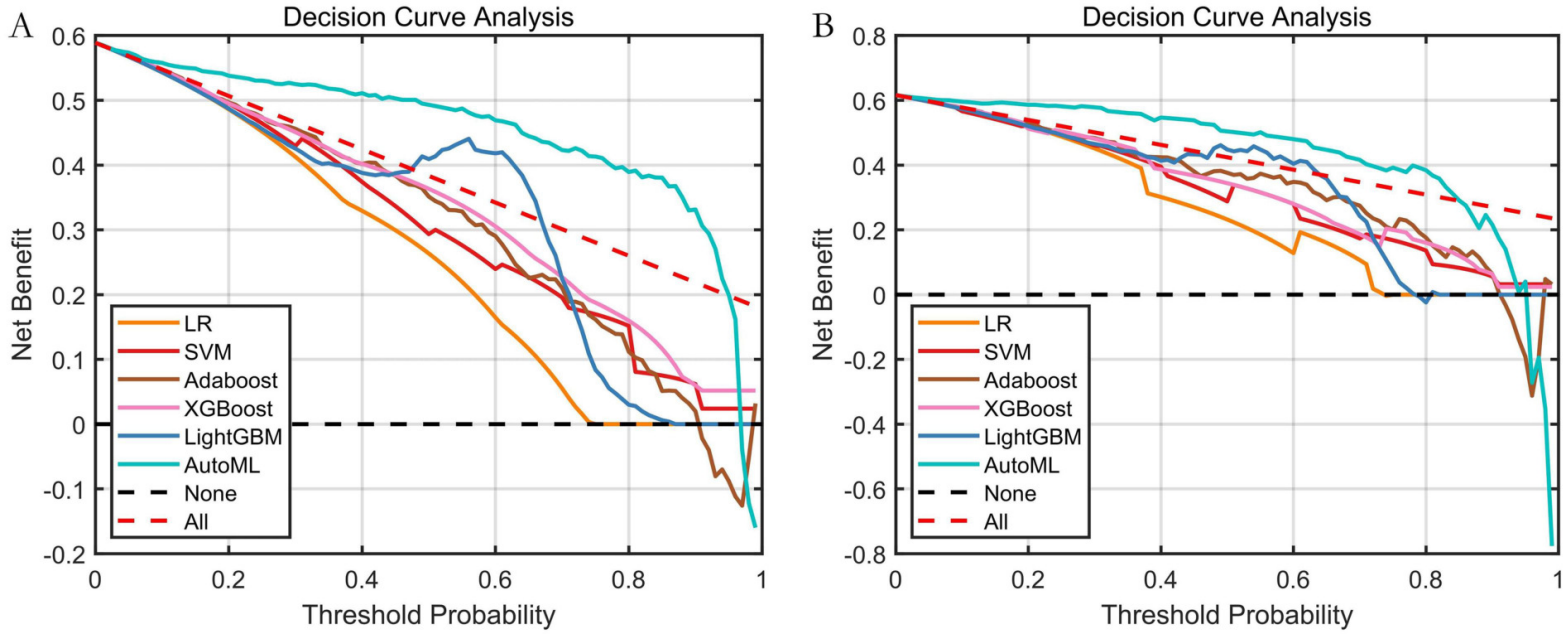
Decision curve analysis (DCA) of predictive models. **(A)** Training set; **(B)** Test set; The *Y*-axis displays net benefit. The solid black line represents the prediction model. The red dashed line represents the “intervene on all” strategy. The black dashed line represents the “intervene on none” strategy.

Decision Curve Analysis (DCA) evaluating the net clinical benefit for predicting symptomatic ICVD risk across different threshold probabilities is shown in Figure 6. DCA curves for the test set clearly demonstrate that utilizing the AutoML model for predicting symptomatic ICVD risk results in a greater net clinical benefit compared to traditional baseline strategies (intervening on all or no patients). This finding underscores the model's significant clinical utility in identifying high-risk patients for targeted intervention.

### 3.6 Feature validation results

#### 3.6.1 LASSO analysis

**Figure 7:**
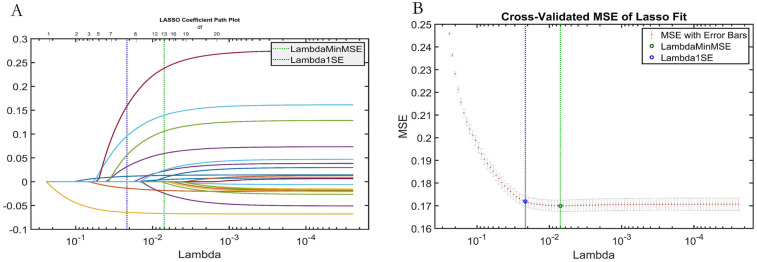
LASSO regression analysis results. **(A)** Variable selection paths (trajectory) plot; **(B)** Cross-validation curve for tuning parameter (lambda) selection.

LASSO regression analysis (Figure 7) was employed for feature selection, providing independent validation of the effectiveness of the features selected by the AutoML model. LASSO identified variables within one standard error of the minimum mean squared error (Lambda1SE) in its sparse model. It selected seven key features: Stenosis Severity, Ulcerative Plaque, Plaque Number, Intraplaque Neovascularization, Hypoechoic Plaque, Age, and Diabetes Status. The overlap rate between the LASSO-selected features and those identified by AutoML was 85.71% (6 out of 7 features), confirming the robustness of the AutoML feature selection.

#### 3.6.2 SHAP analysis

**Figure 8:**
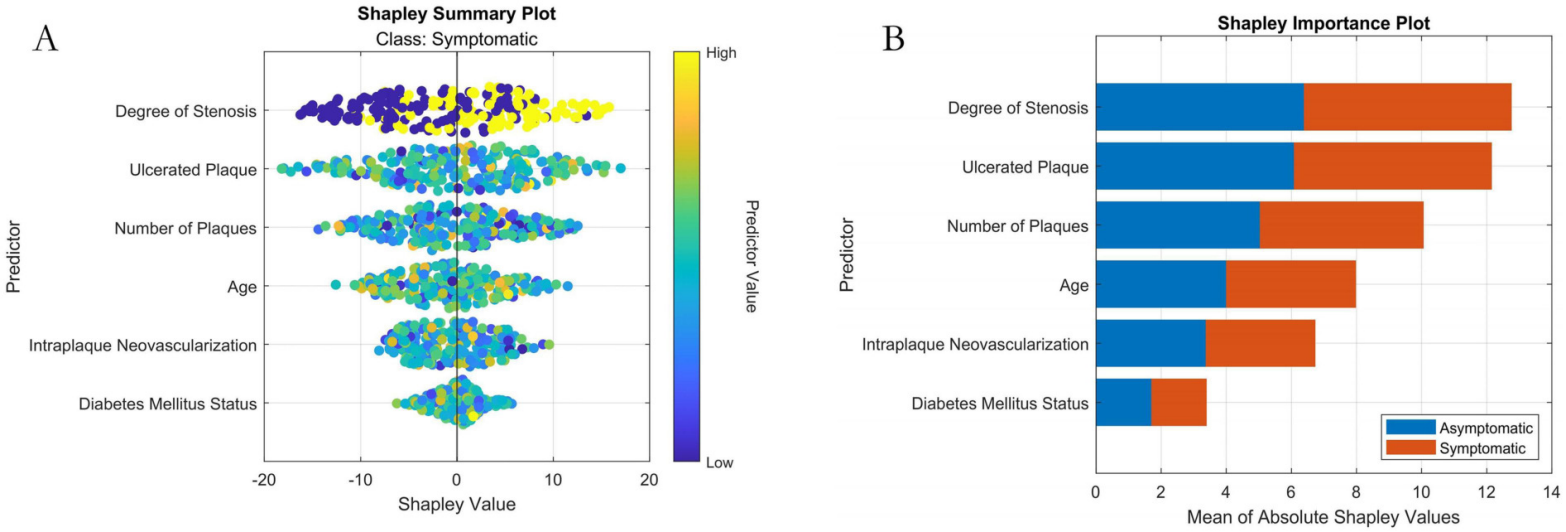
Machine learning interpretability analysis using SHAP. **(A)** SHAP summary plot (Bee-swarm plot visualizing feature contribution per sample); **(B)** SHAP feature importance bar plot.

Based on the results of the SHAP analysis (Figure 8): The ranked feature importance (from highest to lowest contribution to the model's prediction of symptomatic ICVD risk) is: (1) Stenosis Severity; (2) Ulcerative Plaque; (3) Plaque Number; (4) Intraplaque Neovascularization; (5) Age; (6) Diabetes Status. SHAP provides both global insights into overall feature importance (Figure 8B) and local explainability for individual predictions (Figure 8A).

### 3.7 Decision support system development (demonstration)

**Figure 9:**
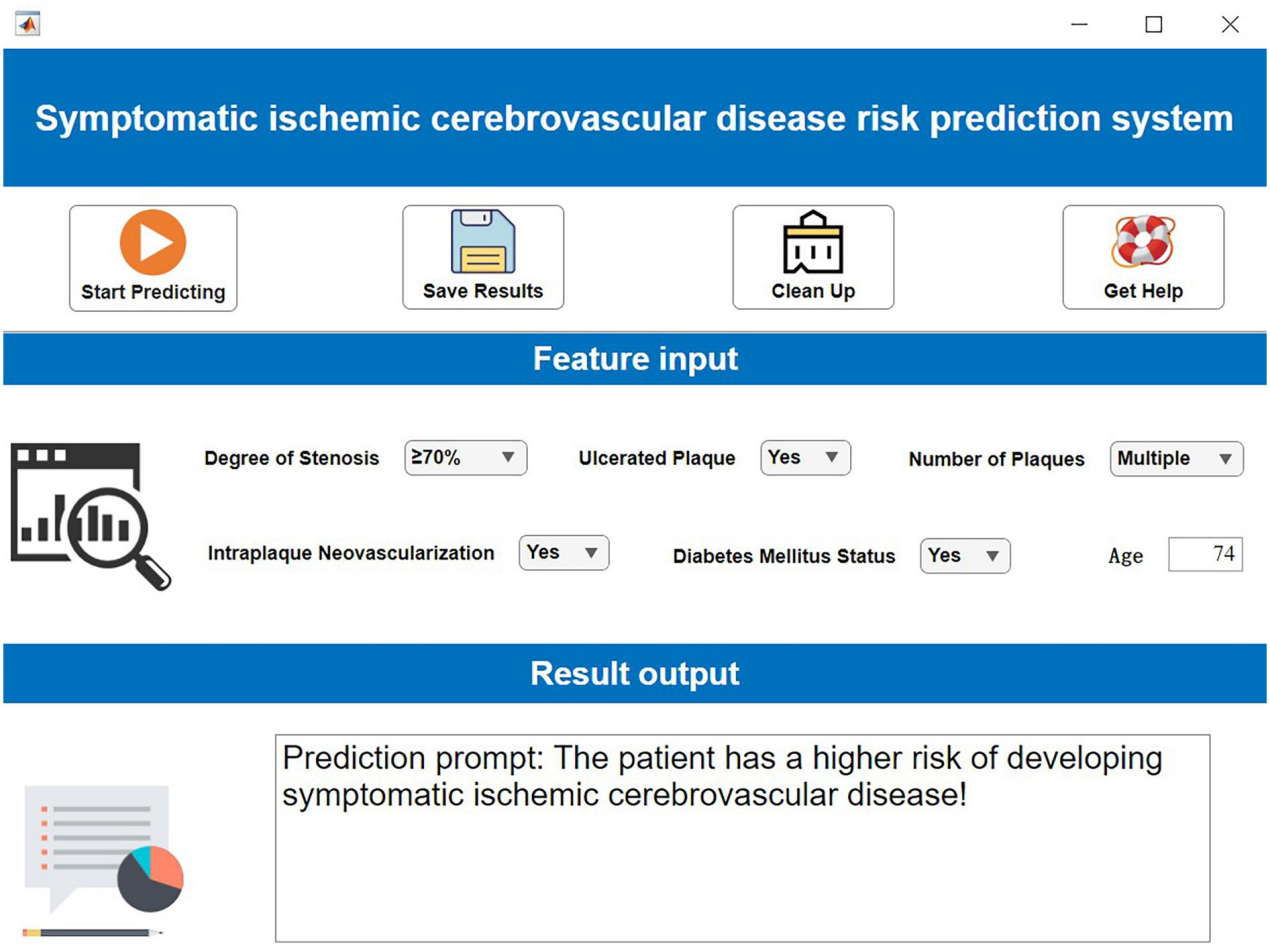
Clinical decision support system demonstration. Created using MATLAB App Designer.

The operational workflow of the developed visualization system is demonstrated in Figure 9. During clinical application: Users simply input the specific values for the required features in the “Feature Input” panel. The system then automatically calculates and outputs the patient's predicted risk of symptomatic ICVD. This provides clinicians with immediate, model-driven risk assessment to support decision-making.

To elucidate the interpretability mechanism of SHAP for individual patient predictions in detail, this study selects a high-risk sample (Patient ID: P-135) for local interpretation. The patient is a 68-year-old male with clinical characteristics including severe carotid artery stenosis (80%), presence of ulcerated plaque, multiple plaques (≥3), intraplaque neovascularization (IPN), and confirmed diabetes. The model predicts a high probability of symptomatic ischemic cerebrovascular events at 0.92. SHAP attribution analysis reveals that ulcerated plaque is the most significant risk driver for this patient (SHAP value +0.38), followed by severe stenosis (+0.35), while the presence of IPN and multiple plaques contribute +0.22 and +0.19 to risk elevation, respectively. Notably, the patient's age of 68 years is below the training set mean age (72.3 years), resulting in a slight protective effect (SHAP value −0.11). From the decision path perspective, the model's base prediction value (i.e., the predicted probability with all features at mean values) is 0.32. Driven strongly by ulcerated plaque and severe stenosis, the risk probability increases substantially. The compounding effects of IPN and multiple plaques further reinforce the high-risk propensity, culminating in a final high-risk determination of 0.92. This outcome is highly consistent with clinical consensus, confirming the importance of ulcer morphology and hemodynamic impairment as core risk factors.

## 4 Discussion

Our study presents an in-depth analysis of a novel risk prediction system for symptomatic ischemic cerebrovascular disease (ICVD) based on an improved machine learning model. The findings unequivocally demonstrate that our developed INRBO-AutoML algorithm model achieves a significant breakthrough in prediction efficacy. Its core value lies in the successful integration of contrast-enhanced ultrasound (CEUS) evaluated intraplaque neovascularization features with multidimensional clinical parameters. Validation on the independent test set revealed the model's exceptional generalization capability, achieving an F1-score of 0.9333, a ROC-AUC (Area Under the Receiver Operating Characteristic Curve) of 0.9343, and a PR-AUC (Area Under the Precision-Recall Curve) of 0.9104. Particularly noteworthy is the performance leap of 22.8% to 29.96% compared to traditional models like Support Vector Machine (SVM, ROC-AUC = 0.766) and Logistic Regression (LR, ROC-AUC = 0.6347). While the model achieved a sensitivity of 100%, theoretically enabling the precise identification of all high-risk patients, it is crucial to acknowledge that no model can achieve completely perfect prediction. This potential limitation might be partially attributable to the current sample size, necessitating future studies with larger cohorts for further validation. Decision Curve Analysis (DCA) further substantiated that applying this model within a specific risk threshold range can lead to a higher net clinical benefit rate compared to conventional strategies. This distinct advantage stems from the model's exceptional capability in successfully integrating complex imaging features—including stenosis severity, ulcerative plaque, and intraplaque neovascularization—as core predictive factors.

Compared with previous research, our work achieves dual breakthroughs. Methodologically, traditional prediction models often rely on single-algorithm architectures, as exemplified by the commonly used Logistic Regression in the Amani study (17), which faces significant limitations in handling high-dimensional heterogeneous data. By introducing the innovative Improved Newton-Raphson-based Optimizer (INRBO), we effectively overcame critical issues such as gradient explosion and initial value sensitivity. Key technical innovations manifested in the application of Tent chaotic mapping to enhance parameter space coverage and the dynamic Gaussian mutation mechanism to accelerate convergence speed. On the application level, early studies like the Sheng trial primarily focused on clinical variables largely overlooking critical image features (18). Our research, however, pioneered the deep integration of intraplaque neovascularization subtypes assessed via contrast-enhanced ultrasound with established clinical characteristics. This novel paradigm of multi-source data integration successfully elucidates complex non-linear associations not previously discovered: LASSO regression identified six core features (including stenosis severity), with an 85.71% overlap rate relative to the AutoML selection. Furthermore, SHAP interpretability analysis revealed stenosis severity as the primary factor in prediction (highest importance ranking). This observation of a graded risk escalation phenomenon provides a crucial theoretical basis for personalized intervention strategies.

The established relevance of the screened factors to symptomatic ICVD occurrence stands as a core discovery of this study. Dual-dimensional validation—through LASSO regression and SHAP interpretability analysis—confirmed the significance of six key predictors: Stenosis Severity, Ulcerative Plaque, Plaque Number, Intraplaque Neovascularization (IPN), Age, and Diabetes Status. These demonstrate well-documented pathophysiological links to disease risk: Stenosis Severity, identified as the primary predictor (SHAP rank #1), primarily stems from hemodynamic alterations (19). Elevated stenosis induces abnormal shear stress that not only activates platelet aggregation pathways leading to thrombus formation but also increases mechanical strain on the fibrous cap. This biomechanical stress potentiates plaque disruption, releasing fragments or thrombi that cause cerebral embolism via distal occlusion. While hypoperfusion remains another important consequence of severe stenosis affecting watershed territories, its contribution to overall stroke risk may be comparatively smaller in populations principally characterized by embolic mechanisms. Ulcerative Plaque and Plaque Number are recognized indicators of morphological instability. While ulceration triggers local inflammation through lipid core exposure, multiple plaque presence signifies systemic atherosclerotic burden that contributes to instability through distinct mechanisms (20). Specifically, plaque multiplicity reflects widespread endothelial dysfunction and vascular inflammation, creating a pro-thrombotic milieu. This amplifies shear stress vulnerability across arterial segments, while simultaneously elevating circulating inflammatory mediators (e.g., IL-6, CRP) that degrade fibrous caps through matrix metalloproteinase activation. Consequently, patients with multiple plaques exhibit substantially higher cumulative rupture risks compared to single-plaque counterparts-a phenomenon consistently demonstrated in longitudinal studies (21). The association of IPN is particularly prominent. Elevated IPN grades are directly linked to VEGF overexpression and MMP-9 secretion, causing fibrous cap degradation and accelerating plaque rupture (22). Prospective studies indicate that IPN is not only an independent predictor of ICVD recurrence but also correlates with systemic vascular events, highlighting its pan-vascular pathologic significance (23). Age, an immutable demographic factor, exhibits a non-linear risk surge in SHAP analysis attributed to multiple mechanisms including reduced vascular elasticity, impaired endothelial repair capacity, and diminished cerebral vasomotor reactivity—with the latter compromising cerebral blood flow autoregulation and independently elevating hypoperfusion-related stroke risk (24). Diabetes status, a traditional metabolic factor, exerts its influence through mechanisms involving oxidative stress and endothelial dysfunction induced by hyperglycemia, cooperatively driving the progression of atherosclerosis (25). The clinical relevance of these factors is corroborated by the SHAP model's global interpretation. Furthermore, the 85.71% feature selection overlap rate achieved by LASSO regression demonstrates the robustness of the chosen feature set. Overall, the selected factors span imaging and epidemiological dimensions, forming a multi-level risk amplifier: stenosis severity provides the hemodynamic mechanical basis, IPN intensifies molecular-level disruption, and metabolic diseases exacerbate systemic inflammation. Through these complex non-linear interactions, they collectively drive ICVD events, thereby providing a scientific foundation for targeted clinical interventions. This synergistic risk amplification framework helps contextualize the null predictive value observed for bilateral carotid intima-media thickness (CIMT) in our cohort: while population-based studies establish CIMT's utility in primary prevention (26), its significance diminishes in established atherosclerosis where vulnerability mechanisms dominate. The plateau effect in vascular remodeling and diminished discriminatory capacity relative to active processes like intraplaque neovascularization explains why comprehensive plaque morphology integrates multiple synergistic pathways to outperform isolated structural metrics in symptomatic ICVD prediction. Future multi-center validation should explore CIMT's potential early-window utility before advanced plaque formation.

Despite the achievements, the study has limitations requiring acknowledgment: Its single-center retrospective design (*n* = 626) results in insufficient external validation scope, particularly regarding regional specificity. Technically, the decision system's reliance on MATLAB hinders widespread adoption, especially in primary care settings. While IPN assessment employed a standardized grading system, the lack of quantitative microbubble perfusion parameters might limit the depth of biological mechanism interpretation. Methodologically, variations in input feature sets—particularly concerning data preprocessing choices such as feature scaling methods, missing value imputation strategies, or inclusion criteria for ultrasound-derived parameters—can significantly impact AutoML model architecture generation and performance characteristics. Future work should prioritize three key directions: Establishing a multi-center prospective cohort (target sample size ≥3,000 patients), incorporating optical coherence tomography (OCT) for enhanced plaque quantification accuracy. Developing an advanced learning framework based on the Transformer architecture to enable cross-center data collaboration and dynamic analysis of CEUS sequences. Constructing a real-time risk alert platform integrated with electronic medical record systems, and conducting cost-effectiveness analyses under different risk stratification scenarios using the validated model.

In summary, the ICVD prediction model established in our study represents an effective integration of radiomics, machine learning, and clinical decision-making. Its core value extends beyond outperforming traditional models in predictive efficacy. More importantly, it pioneers an interpretable, user-friendly, and highly effective precision risk assessment paradigm, thereby providing a novel approach for individualized prevention and management of cerebrovascular disease.

## Data Availability

The raw data supporting the conclusions of this article will be made available by the authors, without undue reservation.
